# Meta-analysis of the therapeutic effects of antibiotic versus appendicectomy for the treatment of acute appendicitis

**DOI:** 10.3892/etm.2014.1584

**Published:** 2014-02-25

**Authors:** ZHI-HUA LIU, CHAO LI, XING-WEI ZHANG, LIANG KANG, JIAN-PING WANG

**Affiliations:** Department of Colorectal Surgery, The Sixth Affiliated Hospital to Sun Yat-Sen University, Guangzhou, Guangdong 510655, P.R. China

**Keywords:** meta-analysis, acute appendicitis, antibiotic, nonoperative, appendicectomy

## Abstract

Appendicectomy has been the gold standard treatment of acute appendicitis for more than a century, while nonoperative therapies, including antibiotics, have acquired increased interest in recent years. The present meta-analysis aimed to compare the therapeutic effects of antibiotics versus appendicectomy for the treatment of acute appendicitis. Medline, Embase and The Cochrane Library databases were searched. Prospective randomized controlled trials that compared antibiotic treatment with surgery were included. The outcomes evaluated included the time of hospital stay, complications and time to work. There were no statistically significant differences between the antibiotic and appendicectomy groups with regard to the time of hospital stay and complications. However, the time to work was significantly longer in the appendicectomy group when compared with the antibiotic group. In addition, the therapeutic effects of antibiotics and appendicectomy were comparable for the treatment of acute appendicitis.

## Introduction

Acute appendicitis is the most common etiology of acute abdomen (1). Appendicectomy has been the predominant treatment of acute appendicitis for more than a century since being introduced by McBurney in the 1880’s and being performed by Grooves in 1883. Since then, the procedure has been standardized among surgeons (2). In 1886, Fitz published a classic study of 247 patients with perforated appendicitis, whereby early appendectomy was advocated (3). As a classic surgical procedure, open appendicectomy is considered to be safe and effective surgery for acute appendicitis that avoids perforation. It is the gold standard treatment of appendicitis due to low morbidity, short length of hospitalization and rare postoperative complications. Prompt appendicectomy is traditionally used for treating acute appendicitis, with the exception of immediate appendicectomy, which may be technically demanding due to the distorted anatomy and difficulties in closing the appendiceal stump as a result of inflamed tissues (4). Exploratory laparotomy often results in an ileocecal resection or a right-sided hemicolectomy due to technical problems or a suspicion of malignancy resulting from the presence of distorted tissues (5).

Nonoperative management has been used for a number of patients. In 1959, Coldrey reported 471 patients who underwent treatment with antibiotics alone and in 1977, Anonymous reported 425 patients who were treated with antibiotics and traditional Chinese medicine (6,7). Previously, nonoperative treatment has played a minor role in treating acute appendicitis. However, it may be used to avoid surgical complications, including small bowel obstruction and negative appendicectomy. Recently, with the development of imaging diagnosis, including computerized tomography and ultrasound, nonoperative therapy for the treatment of acute appendicitis has acquired increasing interest. Investigations into novel and efficient antibiotics have also provided new opportunities for nonsurgical treatment of appendicitis (8). Antibiotic therapy, the main nonoperative therapy, is becoming increasingly important in the treatment of acute appendicitis (9,10). Previous studies have shown that perforated appendicitis in children may be treated with antibiotics (11–13). Furthermore, retrospective studies in adults with perforated appendicitis who were treated conservatively indicated that late recurrences exhibited a mild clinical course (14). However, morbidity and mortality rates remained unsatisfactory for conservatively treated and appendectomized patients.

Randomized controlled trials (RCTs) have been conducted, however, the benefits of appendicectomy versus antibiotic treatment for appendicitis remain in debate. Specific studies have suggested that the surgical approach demonstrates a number of advantages, while other studies have been unable to conclusively identify a significant difference between the two treatments (15,16). Therefore, the aim of the present meta-analysis was to compare antibiotic and appendicectomy treatment for acute appendicitis in cases where surgeons were not limited by technical constraints.

## Materials and methods

### Search strategy

Objectives, search strategy, study selection criteria, data elements, methods for extraction and methods for assessing study quality were defined. Four independent reviewers completed each step in this protocol and resolved disagreements by discussion.

### Literature search

**Figure 1:**
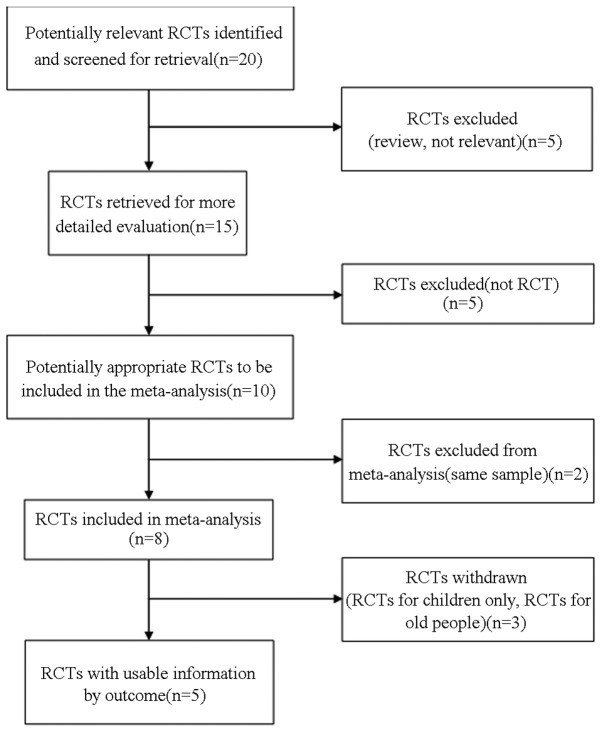
Quality of reporting meta-analyses diagram showing the study methodology. RCT, randomized controlled trial.

To identify all the relevant studies, a computerized search (Medline, http://www.ncbi.nlm.nih.gov/pubmed; Embase, http://www.embase.com/info/helpfiles/; and The Cochrane Library, http://www.thecochranelibrary.com/) was performed using the terms ‘antibiotic’, ‘appendicectomy’, ‘acute appendicitis’, ‘versus’ and ‘conservative’ (Fig. 1). In addition, the reference lists in selected articles were searched manually. There was no language restriction and the time frame was between June 1996 and September 2012. Relevant RCTs were identified that compared antibiotics with surgery for the treatment of acute appendicitis in cases where surgeons were not limited by technical constraints. All patient groups were well matched in terms of subjects and clinical and diagnostic variables at inclusion. Appendicectomy was performed openly or laparoscopically at the surgeon’s discretion. Whenever possible, surgery was performed by a training registrar with an experienced surgeon supervising the operation. Patients in the antibiotic group received intravenous antibiotics for at least 24 h and those whose clinical status had improved the following day were discharged to continue with oral antibiotics for ~10 days. In patients whose clinical conditions showed no improvement, intravenous treatment was prolonged.

### Study selection

Citations selected from the initial search were subsequently screened for eligibility. Diagnosis of appendicitis, determined by the attending physician, was made on the following criteria: History of right lower quadrant pain or periumbilical pain migrating to the right lower quadrant with nausea or vomiting; fever of >38°C or leukocytosis of >10,000 cells/ml; right lower quadrant guarding and tenderness on physical examination; and in certain cases, ultrasonography, computed tomography and gynecological examination. Patients were included in the study if they were aged ≥9 years and were part of a RCT that compared antibiotic treatment with surgery in acute appendicitis. Patients were excluded if they were <9 years old, pregnant, had a history of drug abuse and/or psychiatric disorders and were not involved in a RCTs.

### Data extraction

Data were extracted independently by two reviewers (Liu Zhihua and Qin Huanlong) and cross-checked to reach a consensus. The following variables were recorded: Author, journal, date of publication, geographical region, number of patients, age, gender, body temperature, white blood cell count on admission, C-reactive protein, time of hospital stay, complications and time to work. If necessary, the primary authors were contacted to retrieve further information.

### Statistical analysis

Dichotomous variables were analyzed with odds ratios (ORs) and a fixed-effects model was used according to heterogeneity. Continuous variables, when the mean and SD were presented, were assessed using the weighted mean difference (WMD) and a random-effects model was used according to heterogeneity if significant heterogeneity was present. Sensitivity analysis was applied by removing individual studies from the data set and analyzing the effects on the overall results to identify sources of significant heterogeneity (17). Data analyses were performed using Review Manager version 4.2 software (Nordic Cochrane Centre, Copenhagen, Denmark). P<0.05 was considered to indicate a statistically significant difference.

### Assessment of study quality

Included trials were reviewed and appraised for methodological quality using the Jadad composite scale (17). High-quality trials scored >2 out of a maximum possible score of 5 (18).

## Results

### Description of studies

Of the 983 patients in the five RCTs (19–23), 391 patients were allocated to the antibiotic group, while 592 patients comprised the appendicectomy group. The therapeutic effects of each treatment were evaluated. Patient characteristics and evaluation index are shown in Tables I and II, respectively.

### Methodological quality

The mean Jadad score of the included studies was 3 out of a maximum possible score of 5 (Table III). The main study limitation was associated with the limited sample numbers in three of the studies. However, the sample size of the meta-analysis included 983 patients. Therefore, the limitation may not have an important effect.

### Outcome of comparison

**Figure 2:**
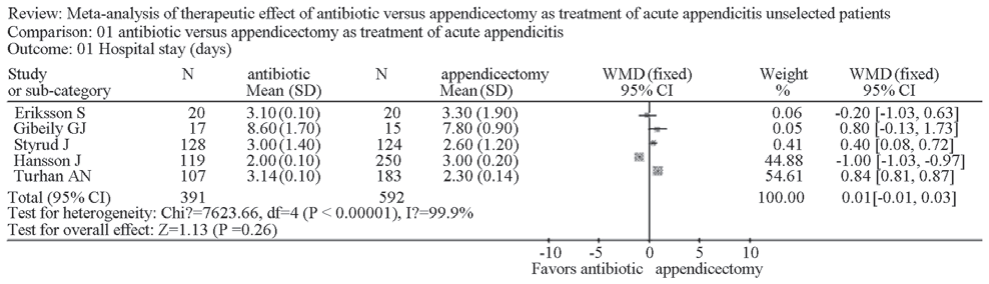
RCTs comparing antibiotic treatment with surgery in acute appendicitis by the time (days) of hospital stay. WMD, weighted mean difference; CI, confidence interval; RCT, randomized controlled trial.

Meta-analysis revealed that the time of hospital stay (days) in the five studies (19–23) was not significantly different in the antibiotic group when compared with the appendicectomy group. In addition, there was no evidence of significant heterogeneity [WMD, 0.01; 95% confidence interval (CI), −0.01–0.03; P=0.26; Fig. 2].

### Complications

**Figure 3:**
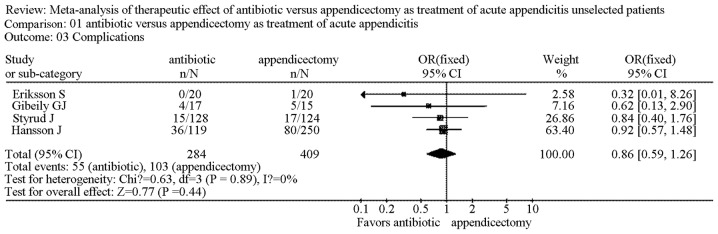
RCTs comparing antibiotic treatment with surgery in acute appendicitis by complications. OR, odds ration; CI, confidence interval; RCT, randomized controlled trial.

Four studies reported complications of the two treatments (19–21,23). Meta-analysis revealed that the complications observed were not significantly different between the antibiotic and the appendicectomy groups (OR, 0.86; 95% CI, 0.59–1.26; P=0.50). In addition, there was no significant heterogeneity (Fig. 3).

### Time (days) to work

**Figure 4:**
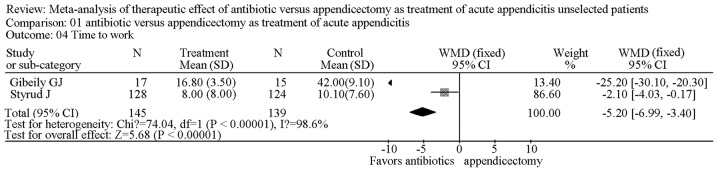
RCTs comparing antibiotic treatment with surgery in acute appendicitis by time (days) to work. WMD, weighted mean difference; CI, confidence interval; RCT, randomized controlled trial.

Two studies reported the time (days) to work (19,25). The results demonstrated that time to work was significantly longer in the appendicectomy group when compared with the antibiotic group (WMD, −5.20; 95% CI, −6.99--3.40; P<0.00001). There was no significant heterogeneity (Fig. 4).

### Sensitivity analysis

Removing individual studies from the data set did not substantially change the OR and WMD values or the level of significance for the three most important clinical outcomes (time of hospital stay, time to work and complications).

### Testing for publication bias

**Figure 5:**
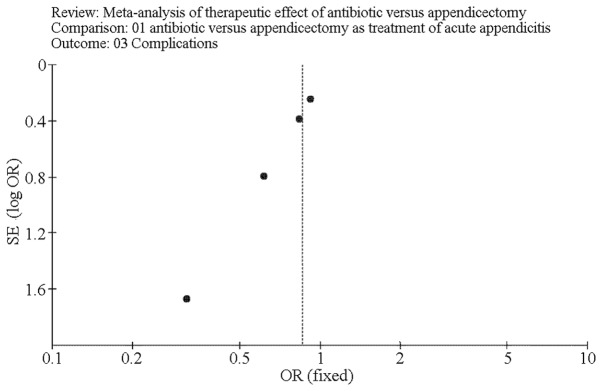
Funnel plot of the complications of antibiotic treatment and surgery. OR, odds ratio.

A funnel plot of the outcome of complications following treatment with antibiotics and appendicectomy in the included studies demonstrated symmetry, indicating there was no serious publication bias (Fig. 5).

## Discussion

Acute appendicitis is the most common intra-abdominal condition requiring emergency surgery (24,25). Appendicectomy has been regarded as the gold standard for acute appendicitis for more than a century. However, conservative treatment has been increasingly studied in selected patients during recent years. In addition, the increasing diagnostic accuracy of acute appendicitis has contributed to the use of antibiotic therapy (26–28).

The present meta-analysis evaluated the therapeutic effects of antibiotics and appendicectomy for the treatment of acute appendicitis. The meta-analysis results indicated that there was no significant difference between the antibiotic and appendicectomy groups with regard to the time of hospital stay and complications. However, time to work was significantly longer in the appendicectomy group when compared with the antibiotic group.

The time of hospital stay was not significantly different between the antibiotic and appendicectomy groups. Conservative therapy has delayed effects on appendicitis compared with that of appendicectomy (29). Therefore, patients must wait for the alleviation of symptoms in hospital. However, following parenteral administration of antibiotics for only 24 h, patients with appendicitis were discharged with oral antibiotics and had a follow-up examination 1 week after discharge, shortening the time of hospital stay (30). Therefore, the present study found that there was no significant differences between the two groups in terms of hospital stay.

There was no significant difference between the antibiotic and appendicectomy groups in terms of complications. However, major complications, including small bowel obstruction, wound rupture and postoperative cardiac problems, were mainly observed in the appendicectomy group, while minor complications, including diarrhea and fungal infection, existed primarily in the antibiotic group (31,32). Therefore, antibiotic therapy is advantageous compared with appendicectomy for the treatment of acute appendicitis (33).

Time to work may be the main advantage of antibiotic treatment for acute appendicitis. Results revealed that time to work was significantly longer in the appendicectomy group when compared with the antibiotic group. This may be due to the longer recovery time required following surgery before patients were able to work (34).

There are additional advantages of antibiotic treatment versus surgery, one of which may be the overall medical cost (35). In addition, antibiotic treatment may avoid surgical complications, including small bowel obstruction and negative appendicectomy (36). Additionally, the rate of mortality is low compared with appendicectomy (37). Antibiotic treatment is also recommended in patients with a high surgical risk, particularly elderly patients with poor heart and lung function or severely obese patients, which may be a contraindication for surgery (21,38).

However, disadvantages of antibiotic treatment also exist. Allergy to the therapeutic agents may withhold the application of antibiotic treatment (39). However, this problem may be avoided relatively easily if surgeons pay increased attention to the possibility of allergies and prescribe antibiotics suitable for different patients (40).

Due to the large number of patients, antibiotic abuse may also be a problem, which may enhance multiple drug resistance in bacterial strains against various antibiotics (41). However, it can be resolved by using antibiotics according to the results of bacterial culture, appropriately. Furthermore, comprehensive medical history and physical examination are necessary and the definite diagnosis may also be confirmed by imaging examination (42).

It is ineffective to analyze trials that differ in terms of underlying conditions and intervention in a meta-analysis (43). Conditions and interventions were similar in the present meta-analysis in order to increase clinical homogeneity between the trials. However, one study discriminated the patient groups as intention-to-treat and per protocol (20) and clinical variables were not mentioned. In addition, one study classified the complications as major or minor (20).

The number of patients is relatively small in numerous RCTs. In the present meta-analysis, a relatively large number of patients were included to enhance the reliability of the results. As previously mentioned, of the 983 patients in the five RCTs, 391 were allocated to the antibiotic group, while 592 comprised the appendicectomy group and the therapeutic effects of the two treatments were evaluated.

In the present study, randomization, allocation concealment and blinding assessment of outcomes were rarely performed, as only two studies were reported as double-blinded tests, due to ethical concern or practical difficulty (20,23). However, this may be superior to a number of other studies, which had rare allocation concealment and blinding assessment (17). Theoretically, the absence of allocation concealment and double-blinding may have resulted in overvaluing the effects of antibiotic treatment on specific measures of postoperative recovery and other associated indexes (44). However, Bruix *et al* reported that individual quality measures, including blinding and allocation concealment, are not reliably associated with the strength of treatment effects in meta-analyses of RCTs (45).

In conclusion, the present meta-analysis indicated that acute appendicitis may be treated successfully with antibiotics. In addition, the therapeutic effects of antibiotics and appendicectomy were comparable for the treatment of acute appendicitis. Therefore, we recommend that more individuals are considered for antibiotic therapy instead of surgery.

## Figures and Tables

**Table I:** Patient characteristics comparing antibiotic treatment with appendicectomy in acute appendicitis.

	Antibiotic treatment/Appendicectomy				
	
Characteristics	Gibeily GJ (14)	Hansson J (15)	Styrud J (16)	Turhan AN (17)	Eriksson S (18)
Patients, n	17/15	119/250	128/124	107/183	20/20
Age, years	38.3	37/34	NR	NR	27.8/35.0
Males, n	40.95/8	62/138	128/124	65/125	14/13
Females, n	12/7	57/112	0/0	42/58	6/7
Body temperature, °C	NR	37.5/37.5	37.5/37.4	NR	37.2/37.1
WBC, k/mm^3^	12.1/14.3	12.2/13.5	12.5/12.4	NR	13.8/13.9
C-reactive protein, mg/l	NR	56/54	55/54	NR	41/40

WBC, white blood cell count; NR, not reported.

**Table II:** Evaluation index comparing antibiotic treatment with appendicectomy in acute appendicitis.

	Antibiotic treatment/Appendicectomy				
	
Index	Gibeily GJ (14)	Hansson J (15)	Styrud J (16)	Turhan AN (17)	Eriksson S (18)
Patients, n	17/15	119/250	128/124	107/183	20/20
Hospital stay, days^a^	8.6±1.7/7.8±0.9	2+0.1/3+0.2	3.0±1.4/2.6±1.2	3.14±0.1/2.4±0.14	3.1±0.1/3.4±1.9
Complications, n	4/5	36/80	15/17	NR	0/1
Time to work, days^a^	16.8±3.5/42±9.1	NR	8.0±80/10.1±7.6	NR	NR

a Values expressed as the mean ± SD.

NR, not reported.

**Table III:** Jadad scores.

	Antibiotic treatment/Appendicectomy				
	
Methodological qualities	Gibeily GJ (14)	Hansson J (15)	Styrud J (16)	Turhan AN (17)	Eriksson S (18)
Was the study described as randomized?	1	1	1	1	1
Was the method used to generate the sequence of randomization described and appropriate?	0	1	0	0	1
Was the study described as double-blind?	0	1	0	0	1
Was the method of double-blinding described and appropriate?	0	1	0	0	1
Was there a description of withdrawals and dropouts?	1	1	1	1	1
Total	2	5	2	2	5

Yes, 1; no, 0; unknown, 0. High-quality trials scored >2 out of a maximum possible score of 5.
